# A Multimaterial Scaffold With Tunable Properties: Toward Bone Tissue Repair

**DOI:** 10.1002/advs.201700817

**Published:** 2018-04-19

**Authors:** Pei Feng, Ping Wu, Chengde Gao, Youwen Yang, Wang Guo, Wenjing Yang, Cijun Shuai

**Affiliations:** ^1^ State Key Laboratory of High Performance Complex Manufacturing College of Mechanical and Electrical Engineering Central South University Changsha 410083 China; ^2^ College of Chemistry Xiangtan University Xiangtan 411105 China; ^3^ School of Energy and Machinery Engineering Jiangxi University of Science and Technology Ganzhou 341000 China; ^4^ State Key Laboratory of High Performance Complex Manufacturing Central South University Changsha 410083 China; ^5^ Key Laboratory of Organ Injury Aging and Regenerative Medicine of Hunan Province Changsha 410008 China

**Keywords:** bioactivity, biodegradability, bone regeneration, cytocompatibility, scaffolds

## Abstract

Polyetheretherketone (PEEK)/β‐tricalcium phosphate (β‐TCP) scaffolds are expected to be able to combine the excellent mechanical strength of PEEK and the good bioactivity and biodegradability of β‐TCP. While PEEK acts as a closed membrane in which β‐TCP is completely wrapped after the melting/solidifying processing, the PEEK membrane degrades very little, hence the scaffolds cannot display bioactivity and biodegradability. The strategy reported here is to blend a biodegradable polymer with PEEK and β‐TCP to fabricate multi‐material scaffolds via selective laser sintering (SLS). The biodegradable polymer first degrades and leaves caverns on the closed membrane, and then the wrapped β‐TCP is exposed to body fluid. In this study, poly(l‐lactide) (PLLA) is adopted as the biodegradable polymer. The results show that large numbers of caverns form on the membrane with the degradation of PLLA, enabling direct contact between β‐TCP and body fluid, and allowing for their ion‐exchange. As a consequence, the scaffolds display the bioactivity, biodegradability and cytocompatibility. Moreover, bone defect repair studies reveal that new bone tissues grow from the margin towards the center of the scaffolds from the histological analysis. The bone defect region is completely connected to the host bone end after 8 weeks of implantation.

## 1 Introduction

Polyetheretherketone (PEEK) is a promising biopolymer, as its mechanical strength and elastic modulus are close to those of human cortical bone.^1^ β‐tricalcium phosphate (β‐TCP), a bioceramic material, has good bioactivity as well as biodegradability.^2^ The binary scaffold composed of PEEK and β‐TCP may theoretically combine the appropriate mechanical properties of PEEK and the good bioactivity and biodegradability of β‐TCP. While in fact PEEK acts as a closed membrane in which β‐TCP is completely wrapped after the melting/solidifying processing, plus PEEK membrane almost does not degrade,^3^ making it difficult for the binary scaffold to exhibit the bioactivity and biodegradability.

How to exert the bioactivity and biodegradability of the binary scaffold consisted of undegradable biopolymer and bioactivity ceramic still remains a challenge. A few researches indicated that blending biodegradable polymer into undegradable polymer could exhibit the biodegradability of scaffolds.^4^ Subramanian et al. had fabricated polyhexylthiophene/poly(lactide‐co‐glycolide) (PLGA) scaffolds via electrospinning and found that the scaffolds possessed good biodegradability due to the addition of biodegradable PLGA.[[qv: 4b]] Some researches had been conducted by blending of biodegradable polymer with bioceramic to display the bioactivity of scaffolds.^5^ Diba et al. had fabricated the polycaprolactone/forsterite scaffolds via salt leaching/solvent casting method and found that the scaffolds exhibited good bioactivity.[[qv: 5a]] The above studies indicated that it may be an effective method to solve the problem by blending a biodegradable polymer with PEEK and β‐TCP.

When the scaffold formed by blending a biodegradable polymer with PEEK and β‐TCP was immersed in body fluid, the biodegradable polymer will first degrade, leaving a lot of caverns on the closed membrane. And then the wrapped β‐TCP may expose from the membrane and exchange ions with body fluid. Among various biodegradable polymers, poly(l‐lactide) (PLLA) has been approved by the United States Food and Drug Administration for use in tissue engineering owing to its good biodegradability and biocompatibility.^6^ It can degrade through hydrolysis of ester groups (COO^−^) in the polymer backbone to lactic acid, which is a normal metabolite of the human body. The lactic acid can finally metabolize to carbon dioxide and water through tricarboxylic acid cycle.^7^ Previous studies have also reported that PLLA possessed good biocompatibility for cell attachment, proliferation and differentiation, and bone regeneration.^8^

In the present study, biodegradable PLLA powders were blended with the PEEK and β‐TCP powders, and PEEK/β‐TCP/PLLA multimaterial scaffolds were fabricated by selective laser sintering (SLS). The biodegradability of the scaffolds was characterized in the form of morphological change and weight loss in phosphate buffered saline (PBS). The bioactivity was assessed by examining apatite formation on scaffold surface in simulated body fluid (SBF). Besides, cytocompatibility was determined by studying the cell behaviors including adhesion, proliferation, and differentiation. Moreover, in vivo bone defect repair capacity of the scaffolds was investigated by radiological examination and histological analysis.

## 2 Results and Discussion

**Figure 1:**
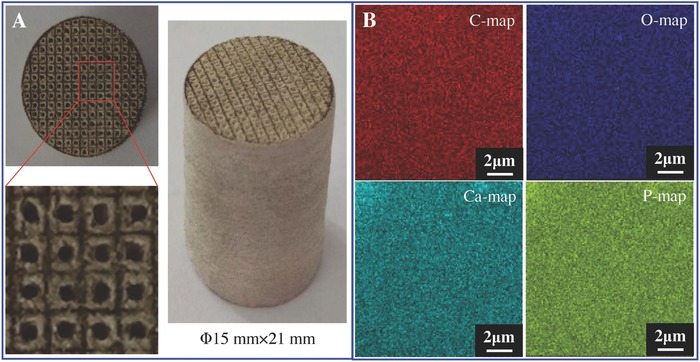
The characteristics of scaffolds. A) Optical graphs. B) EDS mapping images.

The PEEK/β‐TCP/PLLA mixture powders were prepared by adjusting the PEEK and PLLA content while the total content of PEEK and PLLA was kept at 80 wt%. The four diffraction peaks (2θ = 18.7°, 20.7°, 22.8°, and 28.7°) were corresponded to PEEK,^9^ and the characteristic peaks for both PLLA (2θ = 16.3°)^10^ and β‐TCP (2θ = 25.7°, 27.7°, 31.1°, and 34.3°)^11^ were presented, suggesting the presence of both PLLA and β‐TCP in the mixture powders. The PLLA powders and β‐TCP powders were uniformly dispersed throughout the PEEK matrix (Figure S1, Supporting Information). The scaffolds possessed 3D porous structures with dimensions of Φ15 mm × 21 mm fabricated by SLS technology (**Figure** **1**A). The pore sizes and strut sizes of the scaffolds were about 450 and 500 µm, respectively. The mapping distribution of C, O, Ca, and P elements indicated the β‐TCP powders were uniformly distributed in the scaffolds (Figure 1B). The Ca/P ratio was 1.48, which was close to the ratio of 1.5 for β‐TCP.^12^ It has been reported that an average pore size in the range of 300–500 µm of scaffolds is essential for new bone formation.^13^ The pore structure can be fabricated via SLS,^14^ electrospinning,^15^ gas foaming,^16^ particulate leaching,^17^ and so on. Among these, SLS has received considerable attentions because it can easily control the pore size.^18^ In this study, the pore size of the PEEK/β‐TCP/PLLA scaffolds was fabricated at around 450 µm by controlling laser spot diameter and scanning line interval.

**Figure 2:**
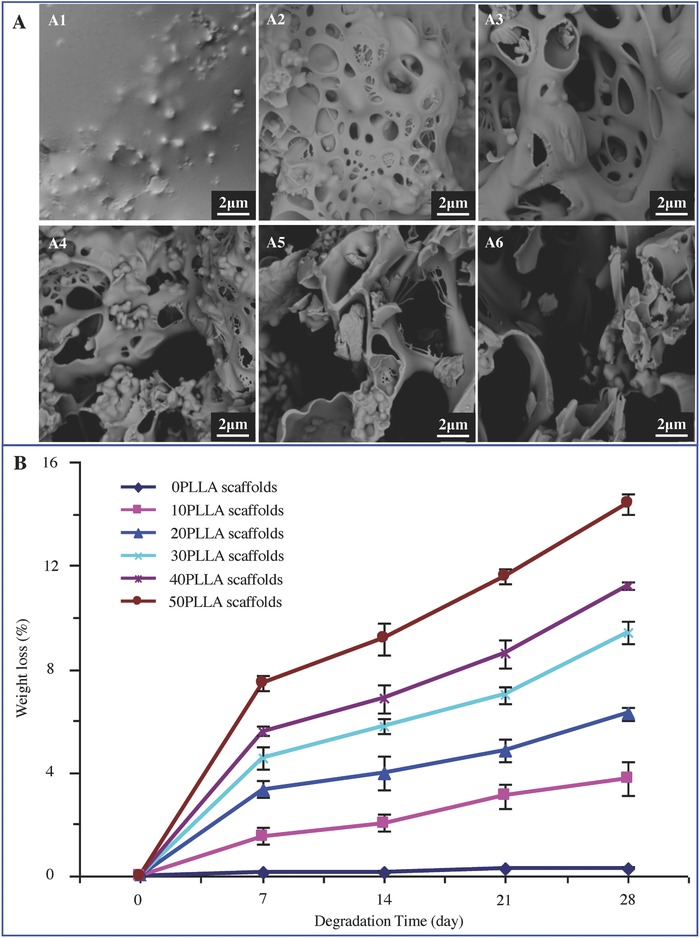
The degradation behaviors of the scaffolds after PBS immersion. A) SEM micrographs of the scaffolds with 0–50 wt% of PLLA content (A1–A6) after PBS immersion for 28 d. B) Weight loss of the scaffolds with 0–50 wt% of PLLA content as a function of degradation time.

The biodegradation behaviors of the scaffolds were characterized in terms of the morphological change and weight loss in PBS at 37 °C. After being immersed for 28 d, the surface morphologies of the scaffolds with 0–50 wt% of PLLA content changed significantly due to PLLA degradation (**Figure** **2**A). The 0PLLA scaffolds had a compact structure (Figure 2A1), and caverns were not found on the surface there. Some small caverns were formed on the 10PLLA scaffolds (Figure 2A2). The cavern size and depth increased rapidly as PLLA content increased. For the 30PLLA scaffolds, some large caverns appeared on the scaffold surface due to the fusion of smaller ones, and the caverns grew deeper and more irregular (Figure 2A4). Most of the smaller caverns disappeared and the larger caverns formed on the 50PLLA scaffolds (Figure 2A6). The scaffold structure was relatively stable as PLLA content increased from 0 to 30 wt%, while parts of the scaffold structure were collapsed as PLLA content further. The weight loss of the scaffolds with 0–50 wt% of PLLA content over a 28‐day period was obtained by gravimetric analysis (Figure 2B). There was no weight loss for the 0PLLA scaffolds, indicating no degradation of the scaffolds. The weight loss of the scaffolds increased with increasing PLLA content.

**Figure 3:**
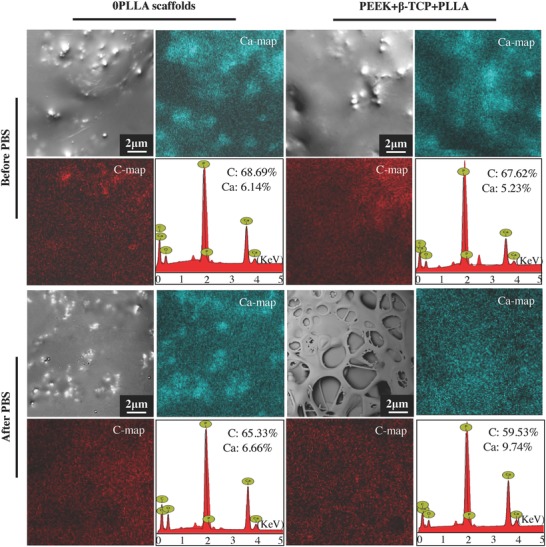
The surface morphologies and the corresponding elemental mapping images of the 0PLLA scaffolds and the scaffolds with PLLA before and after PBS immersion.

The surface morphologies and the corresponding energy dispersive X‐ray spectroscopy (EDS) mapping images of the scaffolds without and with PLLA before and after PBS immersion for 28 d were characterized using scanning electron microscopy (SEM) and EDS analysis (**Figure** **3**), respectively. For the 0PLLA scaffolds, the β‐TCP particles were completely wrapped in the PEEK membrane. The main chemical elements including C, O, Ca, and P were observed from the EDS mapping images. The C element was derived from PEEK, while the Ca and P elements were derived from β‐TCP. The Ca element concentrations in the scaffolds before and after PBS immersion were 6.14% and 6.66%, respectively. For the PEEK/β‐TCP/PLLA scaffolds with PLLA, the surface morphologies changed markedly with lots of caverns after PBS immersion for 28 d. Some of the β‐TCP particles were exposed on the cavern wall due to the PLLA degradation. As a result, the Ca element concentration in the scaffolds increased significantly to 9.74%, which was much higher than that in the scaffolds before PBS immersion.

**Figure 4:**
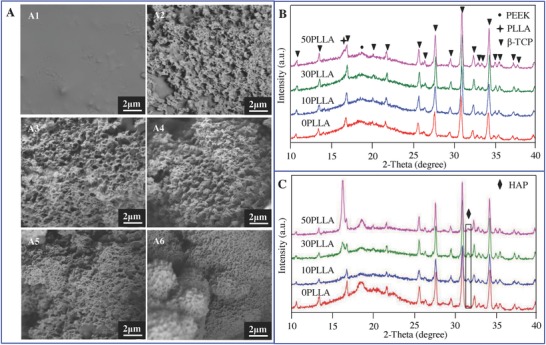
The bioactivity of the scaffolds with 0–50 wt% of PLLA content before and after SBF immersion for 28 d. A) SEM micrographs of the scaffolds (A1–A6) after SBF immersion. B) XRD patterns before SBF immersion. C) XRD patterns after SBF immersion.

The bioactivity was investigated by evaluating the apatite formation on their surfaces in SBF for 28 d, and the corresponding morphologies were observed by SEM (**Figure** **4**A). It could be observed that there are no deposits on the 0PLLA scaffold surface (Figure 4A1), while the number of deposits increased significantly with increasing PLLA content (Figure 4A1–A6). The deposits grew in size and formed a continuous layer on the surface of scaffolds when PLLA content increased to 30 wt% or above. To identify phase compositions of the deposits, X‐ray diffraction (XRD) was conducted on the scaffolds after SBF immersion for 28 d, while the scaffolds without SBF immersion were served as control. Before SBF immersion, the characteristic peaks corresponding to both PEEK and β‐TCP were detected in the 0PLLA scaffolds (Figure 4B). The characteristic peak at 2θ = 16.3° belonging to PLLA appeared and the relative intensity increased with the increasing of PLLA content. There were no other peaks formed in the scaffolds beside the characteristic peaks of PLLA, β‐TCP, and PEEK. These results indicated that no chemical interaction between PLLA, PEEK, and β‐TCP occurred. After SBF immersion, a characteristic peak at 2θ = 31.8° was ascribed to the diffracting plane (211) of hydroxyapatite (HAP),^19^ and the relative intensity increased with increasing PLLA content in the scaffolds (Figure 4C). The results demonstrated that the scaffolds possessed the capability to induce apatite layer formation, and the formation capability could be enhanced as PLLA content increased from 0 to 30 wt% while changed little as PLLA content further increased.

Ideally, scaffold materials should be biodegradable while new bone tissue is growing, and have good bioactivity to promote the bone‐binding ability and osteocompatibility for bone regeneration. Some recent studies have used surface functionalization methods^20^ (such as noncovalent functionalization and covalent functionalization) and biodegradation behavior approaches^21^ (such as copolymerization and blending) to synthesize scaffold materials for tissue engineering applications. Abueva et al.[[qv: 20a]] had fabricated a multichannel hydroxyapatite scaffold, which was functionalized with phosphonic groups using poly(vinyl phosphonic acid) to allow adsorption of a chitosan layer. This modification improved the biocompatibility of the scaffold and also served as a buffer between the implant scaffold and bone tissue. Kundys et al.[[qv: 21a]] had synthesized polylactide (PLA)–poly(1,4‐butylene adipate) (PBA) copolymers and found that the copolymerization of PBA with PLA could adjust the degradation rate of copolymers. It is well known that PEEK is a bioinert material and thereby has limited ability to bind with natural bone tissue.^22^ Introduction of bioceramic (such as TCP, HAP, bioglass, and so on) is an effective method for improving the bioactivity.^23^ In fact, PEEK acts as a closed membrane in which bioceramic is completely wrapped after the melting/solidifying processing, plus PEEK almost does not degrade, hence the scaffolds cannot display the bioactivity and biodegradability. Therefore, in this present study, biodegradable PLLA was introduced to exert the bioactivity of β‐TCP. The results from the biodegradability and bioactivity tests showed that a lot of caverns formed on the closed membrane due to PLLA degradation, and the caverns became larger and deeper with PLLA content increasing. As a result, the wrapped β‐TCP particles exposed from the membrane into body fluid environment. The β‐TCP particles degraded and exchanged ions with body fluid, and hence induced the deposition of apatite on scaffolds surface.

**Figure 5:**
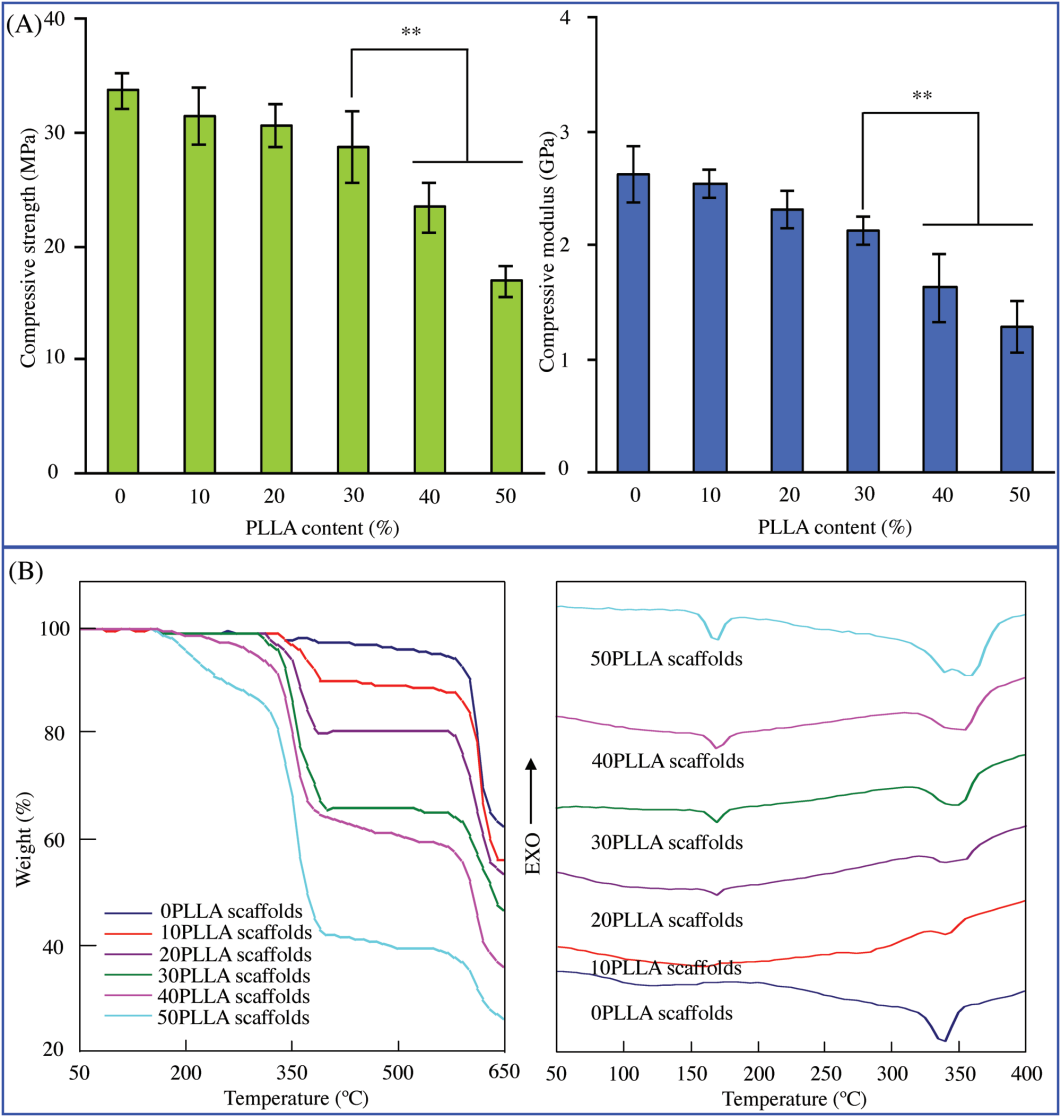
The mechanical and thermal properties of the scaffolds with 0–50 wt% of PLLA content. A) Compressive strength and modulus (**P < 0.01). B) TGA and DSC profiles.

Compression tests were conducted to investigate the compressive strength and modulus at different PLLA content (**Figure** **5**A). It could be seen that the 0PLLA scaffolds had the strong ability to resist compressive deformation with the maximum compressive strength (33.64 ± 1.62 MPa) and modulus (2.62 ± 0.24 MPa). Conversely, the 50PLLA scaffolds had the lowest compressive strength (16.89 ± 1.29 MPa) and modulus (1.28 ± 0.23 MPa). The strength and modulus of other types of scaffolds were intermediate between those of the 0PPLA scaffolds and 50PLLA scaffolds, which exhibited a trend of decline with the increase in PLLA content. Moreover, it was interesting to observe that the strength and modulus of the scaffolds changed relatively stable as PLLA content increased from 0 to 30 wt%, while decreased dramatically as PLLA content further increased. The strength and modulus of the 30PLLA scaffolds were considerably higher than that of the 40PLLA scaffolds and 50PLLA scaffolds (*P* < 0.01). In fact, PEEK possessed excellent strength and modulus, which were much higher than that of PLLA.^24^ As a result, it was reasonable to make a conclusion that incorporation of PLLA into PEEK decreased the mechanical properties of scaffolds, and the mechanical properties were mainly dominated by the continuous phase PEEK in the case of PLLA content less than 30 wt%.

Thermogravimetric analysis (TGA) and differential scanning calorimeter (DSC) analysis were carried out to investigate the thermal properties of the scaffolds at a flow rate of 10 °C min^−1^ (Figure 5B). It could be seen that the scaffolds without PLLA revealed two‐stage thermal decomposition, while the scaffolds with PLLA exhibited three‐stage thermal decomposition from TGA curves. The thermal decomposition of PLLA mainly occurred within a temperature range between 180 and 380 °C.^25^ The stage ranging from 550 to 630 °C was due to the thermal decomposition of PEEK.^26^ The results from TGA analysis indicated that the addition of PLLA in PEEK decreased the thermal stability of scaffolds, as the starting decomposition temperature shifted to lower temperatures. In DSC curves, the endothermic peak at about 334 °C was attributed to the melting temperature of PEEK.^27^ Two endothermic peaks located at about 174 and 355 °C were attributed to the melting temperature and decomposition temperature of PLLA,^28^ respectively. The relative peak intensity increased with increasing PLLA content. The results indicated that both PEEK and PLLA existed in the scaffolds.

**Figure 6:**
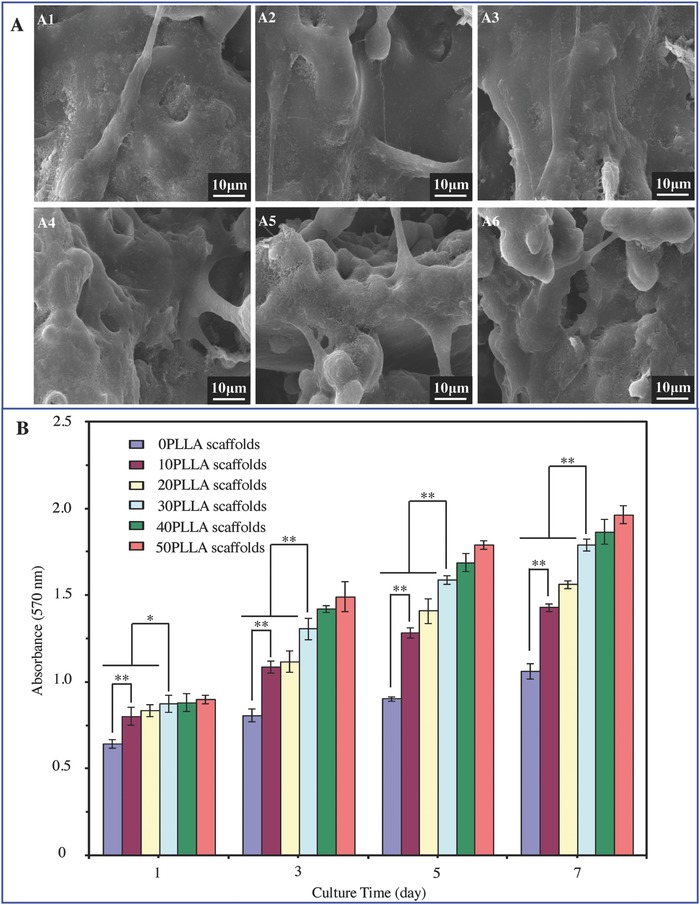
The adhesion and proliferation of cells on the scaffolds with 0–50 wt% of PLLA content. A) SEM micrographs of cells after 7 d of culture. B) Cell proliferation on the scaffolds after cell culture using MTT assay (*P < 0.05, **P < 0.01).

Cell adhesion assay was carried out by seeding MG‐63 cells onto scaffolds and culturing in culture medium for 7 d (**Figure** **6**A). Cells attached tightly on scaffold surface with well‐flattened and expanded morphology. Cells cultured on the PEEK/β‐TCP/PLLA scaffolds with PLLA (Figure 6A2–A6) had well‐spread morphology compared with that on the 0PLLA scaffolds (Figure 6A1). They were fully spread and formed a confluent layer on the surface of scaffolds when PLLA content was 30 wt% or above. The proliferation of MG‐63 cells was investigated by CCK‐8 assay (Figure 6B). It could be seen that all the scaffolds possessed the capability for cell proliferation, and the optical density increased with culture time. Compared with the 0PLLA scaffolds, the PEEK/β‐TCP/PLLA scaffolds with PLLA significantly up‐regulated cell proliferation (*P* < 0.01). The cell proliferation on the scaffolds with 30 wt% or above PLLA content was higher than that on the scaffolds with lower PLLA content (*P* < 0.01 or *P* < 0.05). The differentiation of MG‐63 cells cultured on the scaffolds with 0–50 wt% of PLLA content for 7 d was assessed in terms of alkaline phosphate (ALP) activity (Figure S2, Supporting Information). The ALP activity of MG‐63 cells on the 30PLLA scaffolds was higher than that on the 0PLLA scaffolds, indicating the significant up‐regulated osteogenic differentiation of the cells. The enhanced cell adhesion and accelerated proliferation and differentiation might be related to the improved ions exchange ability between β‐TCP and culture medium due to the PLLA degradation. Previous studies have demonstrated that the released Ca and P ions from bioceramic to culture medium could stimulate cell adhesion, proliferation, and differentiation.^29^

**Figure 7:**
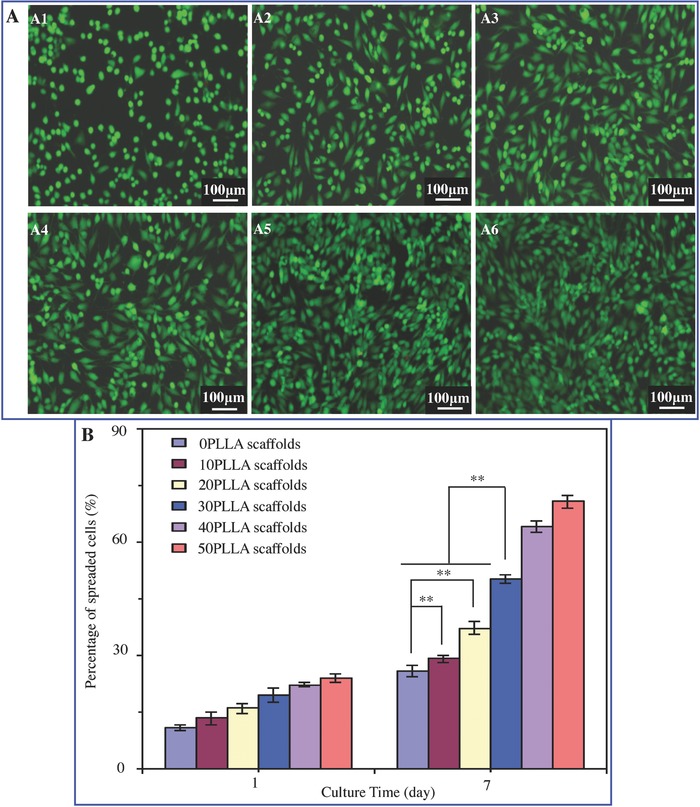
Viability of cells on the scaffolds with 0–50 wt% of PLLA content. A) Calcein‐AM staining images of cells after 7 d of cell culture. B) Percentage of spread cells after 1 and 7 d of cell culture (**P < 0.01).

The viability of MG‐63 cells was assayed using Calcein‐AM staining after 7 d of cell culture, where green fluorescence indicated live cells (**Figure** **7**A). It could be observed that cells spread very well, and a large proportion of live cells adhered on the scaffold surface. The number of live cells increased significantly with increasing in PLLA content in the scaffolds. The percentage of spread cells on the scaffolds was obtained using Photo Shop software (Figure 7B). The number of cells on scaffold surface increased with culture time prolonging. The percentage of spread cells on the 30PLLA scaffolds was significantly higher than that on the scaffolds with 20 wt% PLLA content or below (*P* < 0.01), indicating an improved cell proliferation. The results were consistent with that of the cell adhesion and proliferation assays.

**Figure 8:**
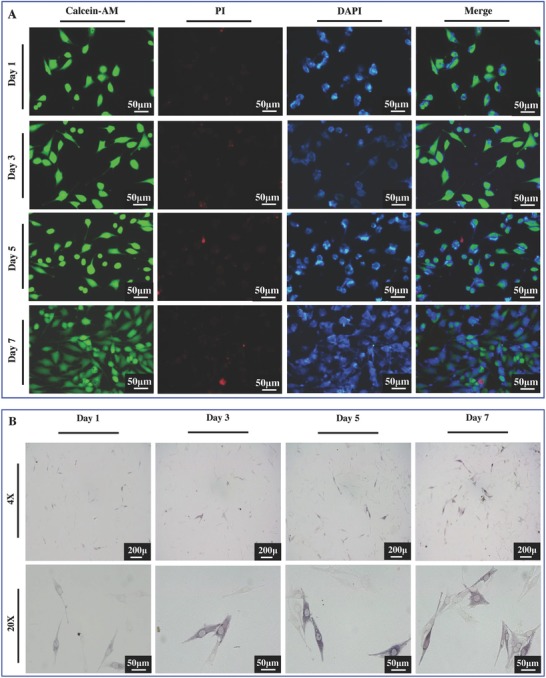
The viability and differentiation of cells on the 30PLLA scaffolds after cell culture for different time intervals. A) Fluorescence morphologies of cells grown on the scaffolds. B) ALP staining images of cells on the scaffolds.

Considering the application for bone regeneration, scaffolds should be biocompatible, mechanically competent, biodegradable, and bioactive. From the above analysis, it was clear that a higher PLLA content in scaffolds led to a higher degradation rate, bioactivity, and cytocompatibility. The mechanical properties of the scaffolds changed relatively stable as PLLA content increased from 0 to 30 wt%, but decreased dramatically as PLLA content further increased due to the collapse of scaffolds. The scaffolds possessed good degradability, bioactivity, and cytocompatibility at PLLA content of 30 wt%. The comprehensive performances of the scaffolds were undoubtedly optimal when PLLA content was 30 wt%. Therefore, the 30PLLA scaffolds were adopt to evaluate the cell viability and differentiation as well as the bone defect repair capacity, while the 0PPLA scaffolds served as control. Live MG‐63 cells were stained with calcein acetoxymethylester (Calcein‐AM, green), dead cells were stained with propidium iodide (PI, red), while cell nucleuses were stained with 4,6‐diamidino‐2‐phenylindole (DAPI, blue) (**Figure** **8**A). Live cell density after 7 d of cell culture was much higher than that after 1 day of cell culture. Very few dead cells were observed for all the culture time points. ALP staining was conducted after cell culture to study the cell differentiation (Figure 8B). From the staining images, it could be seen that the 30PLLA scaffolds had the ability to promote MG‐63 cells differentiation, and the ALP activity increased with increasing culture time.

**Figure 9:**
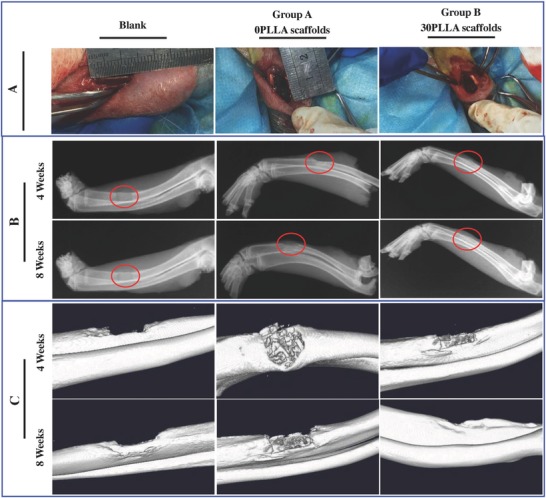
Bone regeneration ability of the scaffolds. A) Surgical procedure for creating bone defect model in rabbits. B) X‐ray images of radius regeneration after 4 and 8 weeks of surgery. C) Micro‐CT images of new bone formation after 4 and 8 weeks of surgery.

Radius bone defect of New Zealand white rabbit was adopt as animal experimental model to evaluate the bone regeneration ability of the scaffolds. The experimental groups A and B were packed with 0PLLA scaffolds and 30PLLA scaffolds, respectively, while the blank group was an untreated defect as control (**Figure** **9**A). No signs of inflammation or infection were observed for any animal after implantation. The rabbits were radiographed using X‐ray to evaluate the degree of bone regeneration after implantation for 4 and 8 weeks (Figure 9B). In the experimental groups A and B, opaque calcified shadow was observed after implantation for 4 weeks, but the calcified density was lower than that of normal tissues. The area of shadow in experimental group A was larger than that in experimental group B, indicating that the 30PLLA scaffolds had good bone formation ability. After implantation for 8 weeks, the shadow in the bone defect region disappeared in experimental group B, and the bone defect region was completely connected to the host bone end. The shadow in the bone defect region remained in the experimental group A. The bone defect in the blank group remained and could not self‐repair. The bone regeneration ability was also studied by microscopic computed tomography (micro‐CT) (Figure 9C). After 4 weeks, there was obvious new bone formation in the defect region implanted with the 30PLLA scaffolds. However, only minimal new bone formation for the 0PLLA scaffolds and the implanted scaffolds could be clearly observed. After 8 weeks, the defect was completely repaired for the 30PLLA scaffolds, while the defect was not repaired for the 0PLLA scaffolds. There was no evidence of bone defect repair for the blank group.

**Figure 10:**
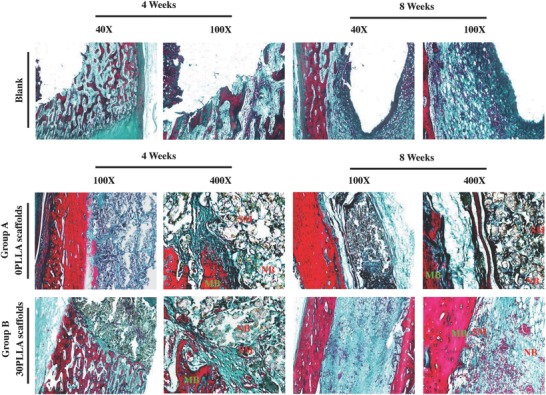
The masson trichrome staining images of bone defect sections in the experimental group A, experimental group B, and blank group after 4 and 8 weeks of surgery (SM: scaffold material; NB: new bone; MB: mature bone).

**Figure 11:**
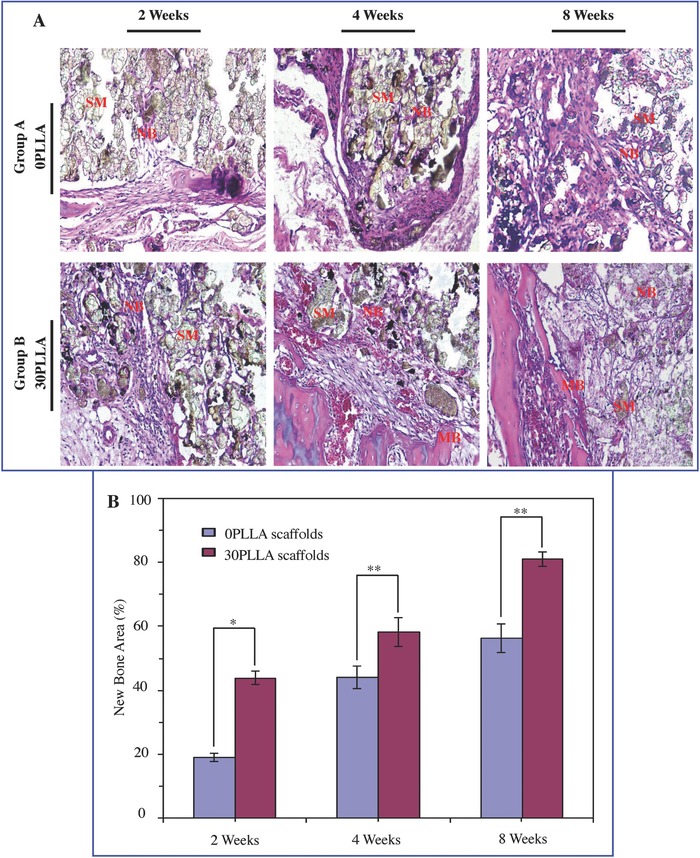
The histological images and quantitative analysis of new bone formation. A) H&E staining images of the bone defect sections in the experimental group A and experimental group B after 2, 4, and 8 weeks of surgery (SM: scaffold material; NB: new bone; MB: mature bone). B) Quantitative analysis of new bone area (*P < 0.05, **P < 0.01).

Histological analysis was performed using Masson trichrome and H&E staining to evaluate the bone defect regeneration quality in all groups. Masson trichrome staining images could give the information on new bone formation after 4 and 8 weeks of surgery (**Figure** **10**). After implantation for 4 weeks, the experimental group B had an obvious increased bone tissue formation as compared with the experimental group A. The new bone tissues grew from the margin toward the center of the scaffolds following with the degradation continued of the scaffold. After 8 weeks, only a small part of scaffold material remained in the bone defect region, and the residual scaffold material formed a coexistence structure with the new bone tissue. The formation ability of new bone tissue in the experimental group B was much higher than that in the experimental group A. While in the blank group, there was almost no new bone tissue formation. H&E staining images of the bone defect sections in the experimental groups A and B after 2, 4, and 8 weeks of surgery were also obtained (**Figure** **11**A). The analysis further confirmed that the 30PLLA scaffolds were able to enhance bone defect repair relative to the 0PLLA scaffolds. The quantitative analysis of the new bone area at each implantation time was calculated from the corresponding H&E staining images (Figure 11B). It could be noted that the new bone area progressively increased over time for all the scaffolds. The amount of new bone tissue in the 30PLLA scaffolds was higher than that in the 0PLLA scaffolds after the same implantation time (*P* < 0.01 or *P* < 0.05). The results indicated that the 30PLLA scaffolds possessed excellent bone formation ability and high efficiency of bone regeneration.

The data from X‐ray and micro‐CT analysis indicated that the 30PLLA scaffolds induced more new bone formation than the 0PLLA scaffolds. And the histological evaluation further confirmed the results. The enhanced bone repair capacity of the 30PLLA scaffolds could be attributed to the degradation of PLLA. It could be observed from the degradation experiments that a lot of caverns formed on the 30PLLA scaffold due to the PLLA degradation. Previous studies had shown that the caverns on the scaffold surface were benefitted to cell migration and proliferation, thus promoting new bone tissue regeneration.^30^ Besides, β‐TCP particles gradually exposed and degraded during the degradation of scaffolds, which provided an ideal microenvironment for osteoblast adhesion, proliferation, and differentiation.^31^ From the cytocompatibility experiments, it had been demonstrated that the 30PLLA scaffolds had enhanced cell adhesion and accelerated proliferation compared with the 0PLLA scaffolds. As a result, the new bone formation ability was significantly improved.

## 3 Conclusion

In the present study, PLLA powders were blended with the PEEK and β‐TCP powders, and the PEEK/β‐TCP/PLLA three‐phase scaffolds were fabricated via SLS for bone regeneration. The scaffolds had good biodegradability with the degradation of PLLA, and lots of caverns formed on the membrane. The wrapped β‐TCP exposed from the membrane and exchange ions with body fluid. As a result, a layer of apatite deposited on the scaffold surface, and the scaffolds displayed good bioactivity. Cell culture experiments demonstrated that the PEEK/β‐TCP/PLLA scaffolds exhibited improved osteoblast adhesion, proliferation, and differentiation, revealing good cytocomptibility. In vivo bone defect repair assessments confirmed that the scaffolds had excellent new bone formation ability and high efficiency of bone regeneration according to the micro‐CT, X‐ray, and histological evaluations. In summary, the PEEK/β‐TCP/PLLA scaffolds possessed good bioactivity, biodegradability, cytocompatibility, and new bone formation capacity, and therefore, have promising potential for bone repair applications.

## 4 Experimental Section

*Fabrication of the Scaffolds*: Mixture powders were prepared by mechanical mixing of PEEK, β‐TCP, and PLLA powders in ethyl alcohol. The PEEK/β‐TCP/PLLA (5:2:3 wt/wt/wt) mixture powders served as an example, and its procedure was described as follows: 2 g of β‐TCP powders (Kunshan Chinese Technology New Materials Co., Ltd, China) were added into 50 mL of ethyl alcohol. The mixture solution was magnetic stirring using a magnetic stirrer (Jintan Ronghua Instrument Manufacture Co., Ltd, China) for 30 min followed by sonication using an ultrasonic cleaning device (Shanghai Kudos Ultrasonic Instrument Co., Ltd, China) for 30 min. Then 5 g of PEEK powders (Dongguan Guanhui Plastic Materials Co. Ltd, China) and 3 g of PLLA powders (average particle size: 0.2–5 µm, purchased from Jinan Daigang Biomaterial Co., Ltd.) were dispersed sufficiently in 150 mL of ethyl alcohol by constant magnetic stirring and sonication. The β‐TCP suspension and the PEEK/PLLA suspension were subsequently mixed and continuously sonicated for another 1 h with magnetic stirring. Thereafter, the mixture powders were collected through filtration, washed with deionized water, and dried at 60 °C in an electrothermal blowing dry box (Guangzhou Dayang Electronic Machinery Equipment Co., Ltd, China). The mixture powders with different PEEK:β‐TCP:PLLA weight fractions of 7:2:1, 6:2:2, 5:2:3, 4:2:4, and 3:2:5 were prepared as described above.

Scaffolds were fabricated using SLS according to the following procedure. Briefly, a cylindrical scaffold model (15 mm in diameter, 21 mm in height) was designed using Solidworks 2011 software (Solidworks Corporation, USA), and then converted into stereolithography (STL) format that could be recognized by the SLS system equipped with a CO_2_ laser (Rofin‐Sinar Laser GmbH, Germany) and a dynamically focusing optical system (Sunny Technology Co., Ltd, China). Laser sintering of the powders was produced using spot diameter of 500 µm, scanning speed of 120 mm s^−1^, scanning line interval of 950 µm, and layer thickness of 0.1–0.2 mm. After sintering was completed, the scaffolds were removed, and unsintered powders were removed by blowing compressed air. The PEEK/β‐TCP/PLLA scaffolds were labeled as 10PLLA, 20PLLA, 30PLLA, 40PLLA, and 50PLLA scaffolds for the 7:2:1, 6:2:2, 5:2:3, 4:2:4, and 3:2:5 ratio of PEEK:β‐TCP:PLLA, respectively. While the PEEK/β‐TCP scaffolds were labeled as 0PLLA scaffolds for the 8:2 ratio of PEEK:β‐TCP as control.

*Characterization of the Scaffolds*: The morphologies were characterized using a SEM (FEI Co., USA). Before SEM observation, the specimens were platinum sputter coated using an auto fine coater (JEOL, Ltd., Japan) under argon atmosphere for 120 s. The elemental distribution of calcium (Ca), phosphorus (P), oxygen (O), and carbon (C) in the scaffolds was investigated using an EDS (Phenom World BV, Netherlands). The phase composition was investigated using a XRD diffractometer (German Bruker Co., German) with Cu Ka radiation (λ = 0.154056 nm, 40 kV, 40 mA). Before analysis, the scaffold specimens were fixed on a specimen holder by double side adhesive tape. The data were recorded in the 2θ range of 10–40° with scanning speed of 8° min^−1^.

Compressive properties were evaluated using a universal testing machine (Shanghai Zhuoji Instruments Co., Ltd, China) at a compression rate of 0.5 mm min^−1^. All scaffolds were prepared in the form of cylinders (Φ15 mm × 15 mm) and vertically placed between two parallel plates. Modulus was computed with the initial slope of the stress–strain curve. Each experiment was repeated six times and the results were averaged. The thermal properties were determined by TGA and DSC using a synchronous thermal analyzer (Nanjing Dazhan Institute of Electromechanical Technology, China) under nitrogen atmosphere. Before measurement, the scaffolds were cut into short pieces, and the specimens with a weight of ≈8 mg were placed in an aluminum crucible. The measurements were carried out over a temperature range of 50–650 °C at a heating rate of 10°C min^−1^. The TGA and DSC data were collected simultaneously during the test.

*Biodegradability and Bioactivity of the Scaffolds*: The biodegradation behaviors of the scaffolds were examined in PBS (Beijing Chemical Reagent Company, China) solution at 37 °C. Five scaffolds (Φ15 mm × 5 mm) for each group were weighed as *W*_0_, followed by immersion in PBS at 37 °C for up to 28 d (7, 14, 21, and 28 d). After completion of each incubation period, the scaffolds were carefully extracted from the medium, rinsed with distilled water for removal of ions absorbed on scaffold surface, dried at 60 °C for 12 h to constant weight, and weighed as *W*_1_. The weight loss (%) was determined using the formula:^32^ Weight loss (%) = (*W*_0_ − *W*_1_)/*W*_0_ × 100%, where *W*_0_ and *W*_t_ are the dry weight of the scaffolds before and after immersion, respectively. The surface morphologies after the scaffolds soaked in PBS at different time intervals were observed using SEM, and the elemental composition and distribution were measured using EDS.

The bioactivity was assessed by immersing the scaffolds in SBF solution at 37 °C for 7, 14, 21, and 28 d and testing the growth of apatite on their surface. The SBF with ion concentrations nearly equal to that of human blood plasma was prepared by dissolving NaCl, KCl, NaHCO_3_, MgCl_2_·6H_2_O, CaCl_2_, K_2_HPO_4_·3H_2_O, and Na_2_SO_4_ (reagent grade, all from Beijing Chemical Reagent Company, China) into distilled water and buffered with trishydroxymethyl aminomethane and hydrochloric acid solution to pH 7.4.^33^ The scaffolds (Φ15 mm × 5 mm) were immersed in the SBF for different time intervals at a concentration of 0.1 cm^2^ mL^−1^ with a volume of 100 mL. After the predetermined time, the scaffolds were removed, rinsed three times with deionized water, and dried at 60 °C for 12 h. The formation of apatite on the scaffold surface was investigated using SEM and XRD.

*Cell Culture of the Scaffolds*: MG‐63 cells (American Type Culture Collection, USA) of passage 3–4 were used to evaluate the cytocompatibility of scaffolds, and grown in Dulbecco's modified Eagle's medium, supplemented with 1 × 10^−3^ m glutamine, 1% penicillin/streptomycin, and 10% fetal bovine serum (all from Cellgro Mediatech, Inc., USA) in a 5% CO_2_ incubator at 37 °C. Before cell seeding, scaffolds (Φ15 mm × 5 mm) were sterilized in 70% ethanol, washed with PBS, and placed under ultraviolet (UV) light for 30 min. Subsequently, the scaffolds were incubated in culture medium for 30 min, and then transferred into 12‐well culture plates. The cell suspension with a density of 1 × 10^5^ cells mL^−1^ was seeded on the scaffolds. The cells/scaffolds constructs were cultured in 95% humidity atmosphere with 5% CO_2_ at 37 °C for up to 7 d (1, 3, 5, and 7 d), and the medium was refreshed every two day.

For cell adhesion, cells/scaffolds constructs were harvested and rinsed with PBS to remove nonadherent cells after culture for a given period. Afterward, they were fixed with 2.5% paraformaldehyde (Sigma‐Aldrich Co., USA), washed with PBS, dehydrated through a series of graded alcohol, and dried in the electrothermal blowing dry box at 40 °C for 24 h. After drying, the specimens were sputter‐coated with platinum and then examined using SEM. For cell viability, the cells/scaffolds constructs were separately exposed to 15 µg mL^−1^ calcein AM, 4.5 µg mL^−1^ PI, and 0.5 µg mL^−1^ DAPI (all from Sigma‐Aldrich Co., USA) for 30 min. The stained specimens were analyzed using a fluorescence microscope (Olympus Corporation, Japan). Stained images were obtained, in which green, red, and blue represented live cells, dead cells, and cell nucleus, respectively. The images were analyzed using Photo Shop software (Adobe Systems Inc., USA) to quantify the percentage of spread cells from six representative images.

For cell proliferation, 20 µL Cell Counting kit‐8 (CCK‐8, Sigma‐Aldrich Co., USA) solution was introduced to each well according to manufacturer's instructions, and then the cells/scaffolds constructs were incubated for 4 h at 37 °C in a CO_2_ incubator after cell culturing. 100 µL of supernatant medium was transferred into a new 12‐well plate, and the optical density at 570 nm of the solution was evaluated using a microplate reader. The ALP activity was evaluated by a colorimetric assay using an ALP reagent with p‐nitrophenyl phosphate (p‐NPP, Sigma‐Aldrich Co., USA) after cell culture. The staining was carried out using ALP staining kit according to manufacturer's recommendations, and the stained scaffolds were photographed using a microscope.

*In Vivo Bone Regeneration*: Bone regeneration ability in vivo was investigated by measuring new bone formation using the model of rabbit radius bone defects. All animal experiments were performed at Xiangya Hospital of Central South University in accordance with protocols approved by the Institutional Animal Care and Use Committee. New Zealand white rabbits at 5 months of age and 2.5–3 kg of weight were randomly divided into three groups corresponding to blank, 0PLLA scaffolds, and 30PLLA scaffolds. Twenty seven rabbits were used for each group and nine rabbits were assigned for different time intervals (2, 4, and 8 weeks). All the rabbits were anesthetized with pentobarbital, and a 20 mm longitudinal incision was made along the radius. After the skin and musculature were separated, a 10 mm bone defect was made using a reciprocating saw. The bone defect modes were established and divided into three groups. Experimental groups A and B were implanted with 0PLLA scaffolds and 30PLLA scaffolds, respectively, while the blank group was kept empty as control. The incision was closed using resorbable suture, and the rabbits were given three days of intramuscular injection of penicillin 10 000 units per day. The rabbits were sacrificed with an overdose of pentobarbital for tissue harvest and analysis after 2, 4, and 8 weeks of surgery.

To evaluate new bone formation in the bone defect sites, the harvested specimens were radiographed using an IVIS Lumina XR instrument (Perkin‐Elmer, USA) at each time point. Radiographs were obtained at a suitable magnification, and the degree of new bone formation was determined by the grey scale from the X‐ray imaging system. For micro‐CT observation, the radius was scanned using a micro‐CT imaging system (SkyScan, Belgium) with 80 kV and 450 µA. After X‐ray and micro‐CT analyses, the harvested bone specimens were fixed in 10% formalin, dehydrated with a graded ethanol series, defatted with chloroform, demineralized with 10% disodium ethylenediaminetetraacetate dihydrate (all from Sigma‐Aldrich Co., USA) solution, and embedded in paraffin blocks. Vertical sections with a 5 µm thickness were cut from the middle of defect using a microtome. They were stained with H&E and Masson's trichrome, and observed using a microscopically. New bone area was measured using the Photo Shop software (Adobe Systems Inc., USA) and calculated by using the following equation:^34^ New bone area (%) = *A*_n_/*A*_o_ × 100%, where *A*_n_ and *A*_o_ are the new bone area and original defect area, respectively. For this analysis, six images were randomly obtained in the same section.

*Statistical Analysis*: All quantitative data were given as mean ± standard deviation unless otherwise stated. Statistical analysis was performed using SPSS 19.0 software (IBM Corporation, USA). Values of *p* < 0.05 were considered significant, while *p* < 0.01 were considered very significant.

## Conflict of Interest

The authors declare no conflict of interest.

## Supporting information

Supplementary
