# Supplementary material for: A Multimaterial Scaffold With Tunable Properties: Toward Bone Tissue Repair
Source: Adv Sci (Weinh). 2018 Apr 19;5(6):1700817. doi: 10.1002/advs.201700817 (PMC6033191; doi:10.1002/advs.201700817)
Supplement: Supplementary file 1 — Supplementary [file ADVS-5-1700817-s001.pdf]

Copyright WILEY-VCH Verlag GmbH & Co. KGaA, 69469 Weinheim, Germany, 2018.

ADVANCED SCIENCE
Open Access

Supporting Information

for *Adv. Sci.,* DOI: 10.1002/advs.201700817

A Multimaterial Scaffold With Tunable Properties: Toward Bone Tissue Repair

*Pei Feng, Ping Wu, Chengde Gao, Youwen Yang, Wang Guo, Wenjing Yang, and Cijun Shuai**

Title: A multi-material scaffold owning tunable properties: towards bone tissue repair

*Pei Feng, Ping Wu , Chengde Gao, Youwen Yang, Wang Guo, Wenjing Yang, Cijun Shuai**

Dr. P. Feng, Dr. C. Gao, Dr. Y. Yang, Dr. W. Guo, Dr. W. Yang, State Key Laboratory of High Performance Complex Manufacturing, College of Mechanical and Electrical Engineering, Central South University, Changsha 410083, China

*Corresponding authors, E-mail: shuai@csu.edu.cn

Prof. C. Shuai

Jiangxi University of Science and Technology, Ganzhou 341000, China

Prof. C. Shuai

State Key Laboratory of High Performance Complex Manufacturing, Central South University, Changsha 410083, China

Prof. C. Shuai

Key Laboratory of Organ Injury, Aging and Regenerative Medicine of Hunan Province, Changsha 410008, China

Dr. P. Wu

College of Chemistry, Xiangtan University, Xiangtan 411105, China

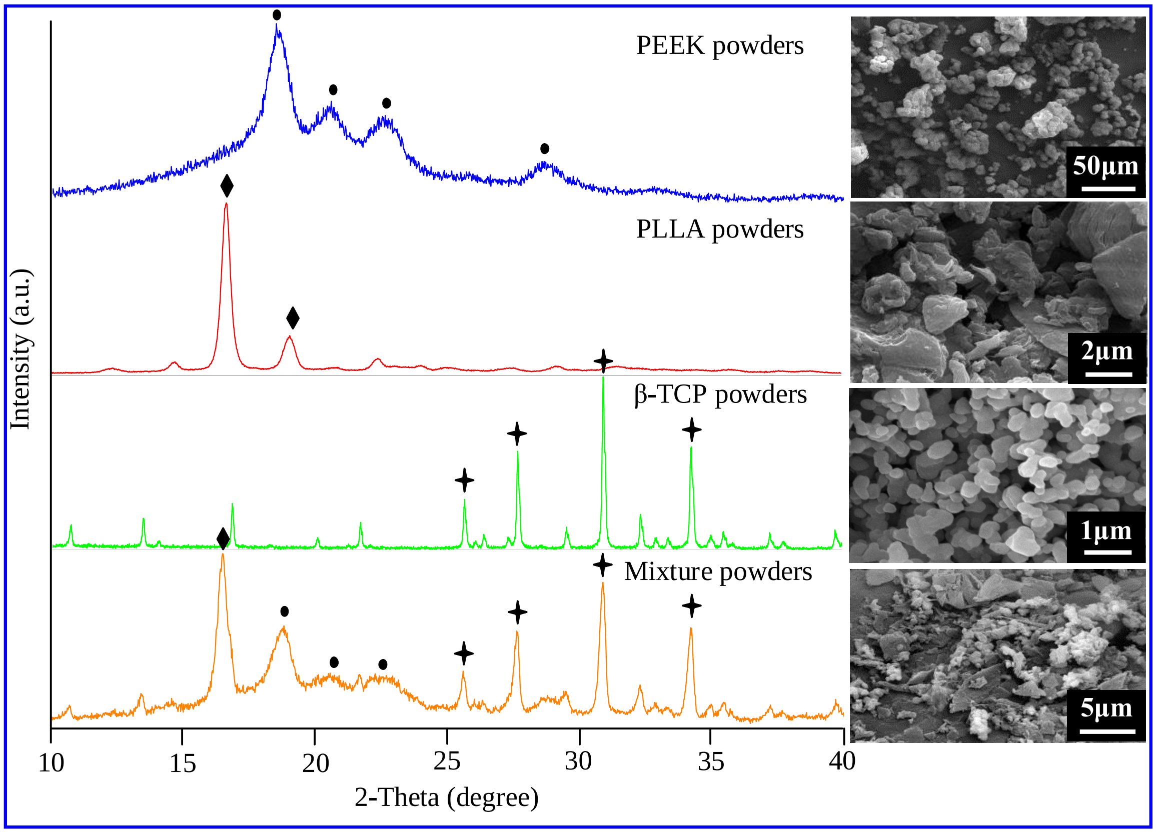

Figure S1. XRD patterns and SEM micrographs of the raw and mixture powders (PEEK:β-TCP:PLLA weight ratio of 5:2:3 was adopt as an example).

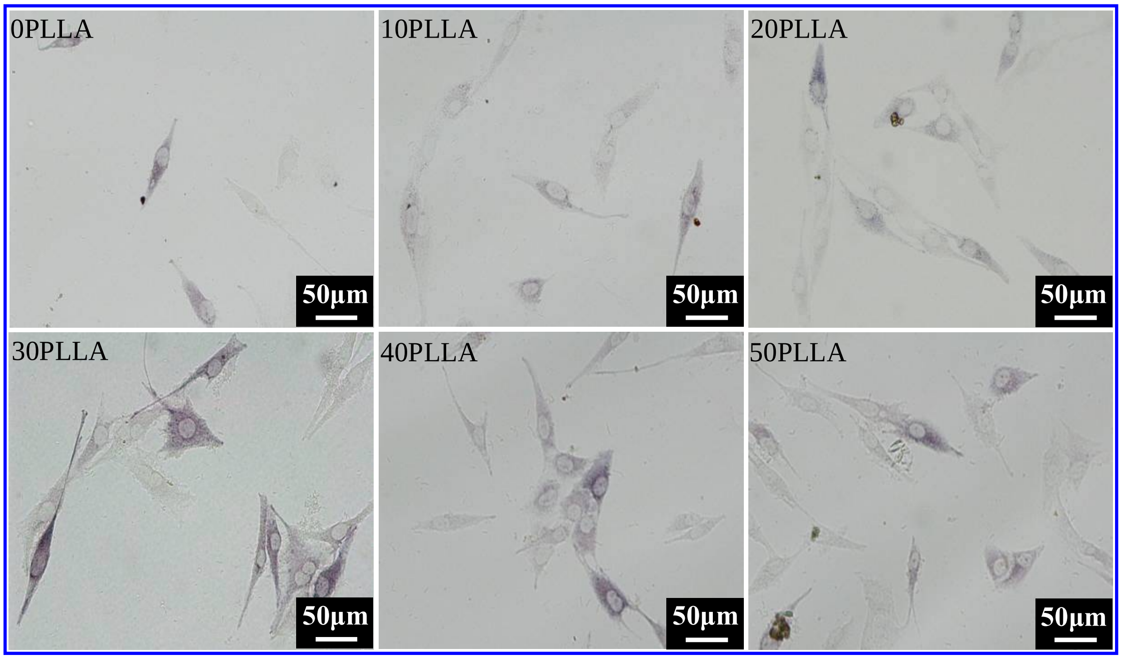

Figure S2. ALP staining images of MG-63 cells cultured on the scaffolds with 0-50 wt% of PLLA content for 7 days.
